# Seasonal dynamics of canine antibody response to *Phlebotomus perniciosus* saliva in an endemic area of *Leishmania infantum*

**DOI:** 10.1186/s13071-018-3123-y

**Published:** 2018-10-11

**Authors:** Rita Velez, Tatiana Spitzova, Ester Domenech, Laura Willen, Jordi Cairó, Petr Volf, Montserrat Gállego

**Affiliations:** 1 ISGlobal, Hospital Clínic - Universitat de Barcelona, Barcelona, Spain; 2 Secció de Parasitologia, Departament de Biologia, Sanitat i Medi Ambient, Facultat de Farmàcia i Ciències de l’Alimentació, Universitat de Barcelona, Barcelona, Spain; 3 Department of Parasitology, Faculty of Science, Charles University, Prague, Czech Republic; 4 Hospital Veterinari Canis, Girona, Spain

**Keywords:** Canine leishmaniosis, *Phlebotomus perniciosus*, Saliva proteins, Markers of exposure, Longitudinal study, North-east Spain

## Abstract

**Background:**

Canine leishmaniosis (CanL) is an important zoonotic parasitic disease, endemic in the Mediterranean basin. In this region, transmission of *Leishmania infantum*, the etiological agent of CanL, is through the bite of phlebotomine sand flies. Therefore, monitoring host-vector contact represents an important epidemiological tool, and could be used to assess the effectiveness of vector-control programmes in endemic areas. Previous studies have shown that canine antibodies against the saliva of phlebotomine sand flies are specific markers of exposure to *Leishmania* vectors. However, this method needs to be further validated in natural heterogeneous dog populations living in CanL endemic areas.

**Methods:**

In this study, 176 dogs living in 12 different locations of an *L. infantum* endemic area in north-east Spain were followed for 14 months. Blood samples were taken at 5 pre-determined time points (February, August and October 2016; January and April 2017) to assess the canine humoral immune response to whole salivary gland homogenate (SGH) and to the single salivary 43 kDa yellow-related recombinant protein (rSP03B) of *Phlebotomus perniciosus*, a proven vector of *L. infantum* naturally present in this region. Simultaneously, in all dogs, *L. infantum* infection status was assessed by serology. The relationship between anti-SGH and anti-rSP03B antibodies with the sampling month, *L. infantum* infection and the location was tested by fitting multilevel linear regression models.

**Results:**

The dynamics of canine anti-saliva IgG for both SGH and rSP03B followed the expected trends of *P. perniciosus* activity in the region. Statistically significant associations were detected for both salivary antigens between vector exposure and sampling month or dog seropositivity to *L. infantum*. The correlation between canine antibodies against SGH and rSP03B was moderate.

**Conclusions:**

Our results confirm the frequent presence of CanL vectors in the study area in Spain and support the applicability of SGH- and rSP03B-based ELISA tests to study canine exposure to *P. perniciosus* in *L. infantum* endemic areas.

## Background

*Leishmania infantum* (Kinetoplastida: Trypanosomatidae) is the causative agent of canine leishmaniosis (CanL), a zoonotic vector-transmitted disease widespread in the Mediterranean region, as well as in other parts of the world [1–3]. Prevalence of *L. infantum* infection in canine populations from endemic areas is highly heterogeneous [4], and not all infected dogs will ever develop clinical signs of the disease [5]. However, infected asymptomatic dogs could act as a reservoir of the parasite and are capable of transmitting *L. infantum* to other dogs, as well as to humans [6, 7].

The transmission of the parasite is mainly vectorial, through the bite of phlebotomine sand flies. In the Mediterranean basin, eight species of the genus *Phlebotomus* have been implicated as vectors of *L. infantum*, according to conventional criteria. From these, all except one belong to the subgenus *Larroussius* [8]. In Spain, CanL transmission is mainly shared by *P.* (*L.*) *perniciosus* and *P.* (*L.*) *ariasi* [9, 10], with the second species having a narrower distribution but being responsible for maintaining the infection at higher altitudes [11, 12]. Recently, *L. infantum* DNA was also found in another *Larroussius* species, *P. langeroni*, in the south of the country [13].

The detection of anti-sand fly salivary antibodies in the blood of vertebrate hosts has proven to be highly specific [14] and was successfully used as a marker of exposure to *L. infantum* vectors [15, 16]. In CanL endemic areas, monitoring the canine IgG response to sand fly saliva can be a useful epidemiological tool [15, 17], complementing studies of vector population dynamics and host-vector interactions, as well as enabling the assessment of risk of *Leishmania* infection [14, 18, 19]. Furthermore, it can be used to measure the effectiveness of vector-control programmes and to assist in the design of better control strategies for the disease [20, 21].

Originally, sand fly whole salivary gland homogenates (SGH) were used to investigate the presence of anti-sand fly saliva antibodies in vertebrate hosts [20–22]. However, its use in large-scale studies is impaired by technical limitations [23]. Additionally, the use of SGH in vector exposure tests may reduce the specificity of detection due to a possible cross-reactivity with saliva of sympatric and closely related sand fly species [24].

An alternative to the use of SGH is the identification of species-specific salivary proteins that can be expressed in recombinant forms and produced in large quantities for use in large-scale epidemiological studies [25, 26]. Recent studies identified *P. perniciosus* yellow-related protein rSP03B as the most promising candidate to replace SGH in the detection of host markers of exposure to this vector species [16, 17, 26]. This recombinant protein has been tested and validated in dogs and other animals in cross-sectional studies [16, 26], as well as in a canine longitudinal study [17], but no information exists on the seasonal dynamics of either SGH or rSP03B in natural heterogeneous dog populations from endemic areas.

Therefore, the objectives of this study were (i) to investigate the dynamics of *P. perniciosus* and their relative density in a previously uncharacterized CanL endemic area through the detection of anti-saliva IgG in dogs; and (ii) to evaluate the performance of both SGH and rSP03B antigens as markers of exposure to *P. perniciosus* in natural canine populations.

## Results

### Seasonal dynamics of IgG response against salivary proteins from *P. perniciosus*

**Fig. 1:**
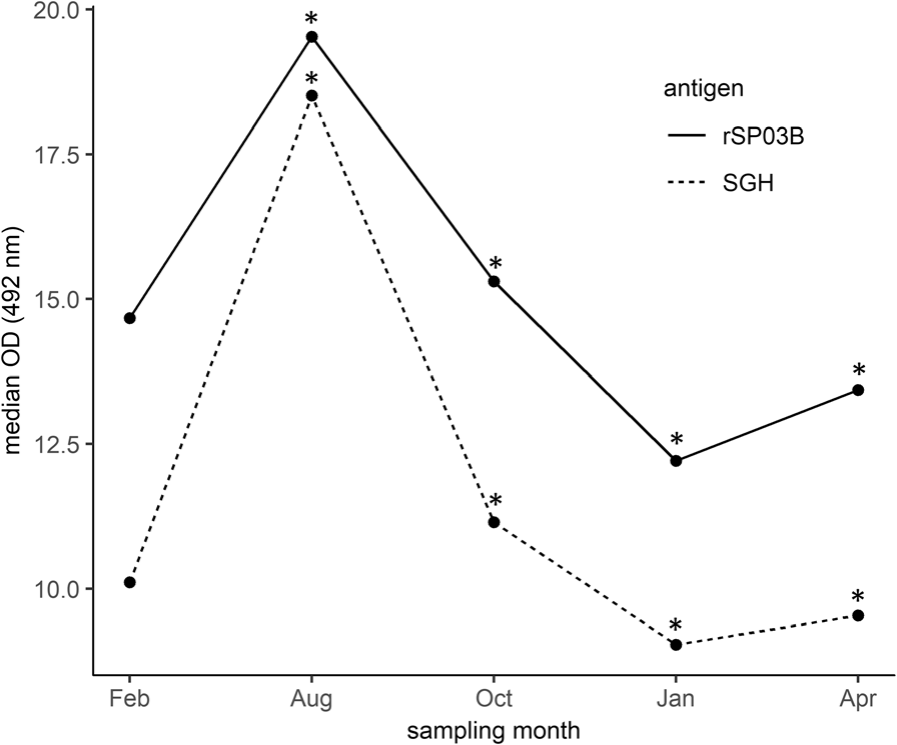
Dynamics of anti-*P. perniciosus* salivary proteins IgG response in dogs from an endemic area during a sand fly activity season. Values presented refer to the normalized OD medians obtained at each sampling month for all dogs and locations. Statistically significant differences in median OD between two consecutive months are marked with an asterisk (*P* < 0.05)

Median values of normalized ELISA OD values for SGH ranged from 9.04 (range: 3.94–66.23) in January 2017 to 18.51 (7.93–100.58) in August 2016 (Table 1). For rSP03B, median OD values varied between 12.21 (6.75–53.71) and 19.53 (10.64–124.01) in January 2017 and August 2016, respectively. With both antigens, median OD readings raised from basal values in February 2016 (10.11 and 14.67 for SGH and rSP03B, respectively) to peak in August (18.51 and 19.53 for SGH and rSP03B, respectively), sustained higher readings in October (11.15 and 15.31 for SGH and rSP03B, respectively), and descended again to basal levels in January (9.04 and 12.21 for SGH and rSP03B, respectively) and April 2017 (9.54 and 13.44 for SGH and rSP03B, respectively). Median normalized ELISA OD results obtained per month for both SGH and rSP03B are described in Table 1 and plotted in Fig. 1.

**Table 1:** Median values of normalized OD readings for SGH and rSP03B obtained per sampling month in all locations

Variable	*N*	SGH	rSP03B
		Median (Range)	Median (Range)
February 2016	174	10.11 (5.49–49.62)	14.67 (7.36–41.24)
August 2016	33	18.51 (7.93–100.58)	19.53 (10.64–124.01)
October 2016	164	11.15 (5.56–86.44)	15.31 (6.15–112.54)
January 2017	154	9.04 (3.94–66.23)	12.21 (6.75–53.71)
April 2017	148	9.54 (5.25–62.59)	13.44 (6.27–36.22)

*Abbreviation*: *N* number of dogs sampled per sampling month

Cut-off values were set at 13 for SGH and 22 for rSP03B. When these were applied to the OD readings obtained in August 2016, 75.76% (25/33) of the dogs were positive to anti-SGH IgG, and 36.36% (12/33) to anti-rSP03B antibodies. In October, these values dropped to 35.98% (59/164) for SGH and 18.9% (31/164) for rSP03B. During the non-transmission season (considered to extend from November to May), the percentage of seropositive dogs ranged from 14.29% (25/175) in February 2016 to 17.57% (26/148) in April 2017 for SGH and 8.44% (13/154) in January 2017 to 12.16% (18/148) in April 2017 for rSP03B.

**Fig. 2:**
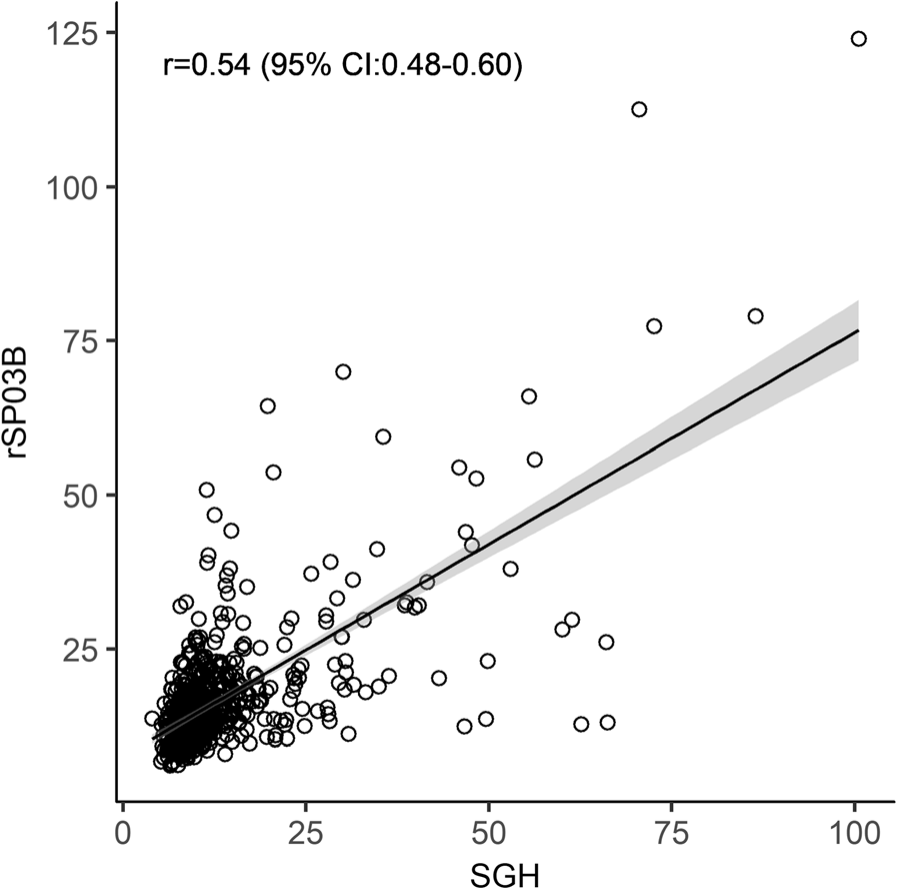
Correlation between IgG recognizing SGH and rSP03B protein in dogs naturally exposed to *P. perniciosus*. Results from both SGH and rSP03B are presented in normalized OD (*r*_*S*_ = 0.54; 95% CI: 0.48–0.60, *P* < 0.001)

Correlation results for IgG response between SGH and rSP03B were *r*_*S*_ = 0.54 (95% CI: 0.48–0.60, *P* < 0.001) (Fig. 2).

### Dogs’ exposure to *P. perniciosus* in the study area

**Fig. 3:**
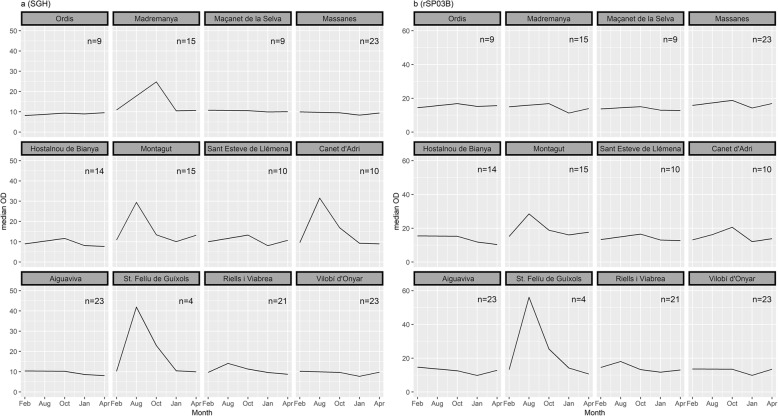
Dynamics of dogs’ IgG recognizing SGH (**a**) and rSP03B protein (**b**) in the different sampling locations during a sand fly activity season. Values presented refer to the normalized OD medians obtained at each sampling month

Exposure of dogs to phlebotomine vectors showed some variation according to the location. Median OD readings varied from 9.11 (range: 5.25–20.57) to 14.14 (7.44–55.45) for SGH ELISA and from 12.71 (7.53–64.44) to 17.87 (8.39–112.54) for rSP03B. Minimum median values of response to both SGH and rSP03B corresponded to the same location (Aiguaviva), but maximum median values were registered in different sites for each antigen (Sant Feliu de Guíxols for SGH and Montagut for rSP03B) (Table 2). Figure 3 presents the dynamics of dogs’ IgG response to SGH (Fig. 3a) and rSP03B (Fig. 3b) in each locality.

**Table 2:** Median values of normalized OD readings for SGH and rSP03B obtained per sampling location at all time points

Variable	*n* (Range)	Geographical coordinates	SGH	rSP03B
			Median (Range)	Median (Range)
Ordis	8 (7–9)	42°13'37.7"N, 2°54'24.1E	9.14 (6.45–45.95)	15.16 (8.35–54.50)
Madremanya	14 (12–15)	41°58'47.0"N, 2°58'7.2"E	11.22 (6.79–49.84)	14.49 (8.95–43.99)
Vidreres	8 (7–9)	41°47'27.4"N, 2°45'0.4"E	10.59 (7.80–16.86)	13.46 (8.58–40.23)
Massanes	21 (20–23)	41°45'15.3"N, 2°38'44.0"E	9.31 (5.67–62.59)	16.35 (7.82–55.81)
Hostalnou de Bianya	12 (11–14)	42°13'26.0"N, 2°26'9.7"E	8.75 (5.35–33.16)	13.19 (6.27–46.82)
Montagut	13 (7–15)	42°14'7.7"N, 2°35'57.6"E	12.01 (3.94–72.61)	17.87 (8.39–112.54)
St. Esteve de Llémena	9 (9–10)	42°3'35.1"N, 2°37'1.4"E	9.49 (6.23–22.40)	14.18 (9.12–22.46)
Canet d'Adri	8 (4–10)	42°1'53.7"N, 2°44'15.3"E	10.61 (6.52–100.58)	14.03 (7.36–124.01)
Aiguaviva	19 (16–22)	41°54'27.2"N, 2°46'19.0"E	9.11 (5.25–20.57)	12.71 (7.53–64.44)
St. Feliu de Guíxols	4	41°47'2.3"N, 2°59'58.7"E	14.14 (7.44–55.45)	16.73 (8.57–65.97)
Riells i Viabrea	20 (18–21)	41°43'59"N, 2°33'39.3"E	10.02 (6.07–66.23)	13.43 (8.59–35.31)
Vilobí d'Onyar	23 (22–23)	41°53'3.2"N, 2°43'38.6"E	9.13 (5.17–16.49)	13.05 (6.15–38.07)

*Abbreviation*: *n* mean number of dogs sampled in each location

The percentage of anti-sand fly saliva seropositive dogs per location, defined as the number of dogs that showed a positive IgG titre at least once during the study period, ranged from 13.33% (1/8) in Ordis to 100% in Canet d’Adri (8/8) and Sant Feliu de Guíxols (4/4) for SGH, and from 8.16% (1/12) in Hostalnou de Bianya to 100% (4/4) in Sant Feliu de Guíxols for rSP03B. Total anti-sand fly saliva seropositivity calculated for the study area was 49.43% (87/176) for anti-SGH IgG and 28.98% (51/176) for anti-rSP03B antibodies.

### Dogs’ exposure to *P. perniciosus* and *L. infantum* infection

Correlation results between antibody response to *P. perniciosus* saliva and *L. infantum* were low both for SGH (*r*_*S*_ = 0.27, 95% CI: 0.19–0.35, *P* < 0.001) and rSP03B protein (*r*_*S*_ = 0.25, 95% CI: 0.18–0.32, *P* < 0.001).

### Multilevel analysis of the relationship between anti-*P. perniciosus* salivary proteins, month and location and *L. infantum* seropositivity

The multilevel model results confirmed the annual dynamics of anti-salivary proteins IgG responses. When compared to the first sampling month (February 2016), IgG responses to SGH significantly rose in August (*t* = 8.55, *df* = 491, *P* < 0.001) and October (*t* = 6.49, *df* = 491, *P* < 0.001) and dropped in January (*t* = -2.49, *df* = 491, *P* = 0.013) and April 2017 (no significant difference when compared to February 2016). As expected, the highest log OD estimate was observed in August 2016 and the lowest in January 2017 (Table 3). The same trend was observed in the model run for the rSP03B protein, with comparable levels of significance (Table 4). There were no significant differences in IgG responses for both antigens between each sampling location and the one set as reference, except for Montagut, where significantly higher OD levels were observed for SGH (*t* = 2.28, *df* = 166, *P* = 0.024) and rSP03B (*t* = 2.13, *df* = 164, *P* = 0.035). According to the multilevel model, seropositivity to *L. infantum* proved to be associated with a rise in anti-salivary proteins OD values for both SGH (*t* = 2.5, *df* = 491, *P* = 0.013) and rSP03B (*t* = 2.15, *df* = 493, *P* = 0.032).

**Table 3:** Estimates of the multilevel linear regression model of the relationship between log transformed normalized SGH OD values and sampling time, location and dog seropositivity to *L. infantum*. “Dog” was included as a random effects variable

Variable	Levels	Estimate	SE	*P*-value^a^
Intercept		2.40	0.06	<0.001
Sampling month	February 2016	Ref	–	–
	August 2016	0.54	0.06	<0.001
	October 2016	0.20	0.03	<0.001
	January 2017	-0.06	0.03	0.013
	April 2017	-0.01	0.03	0.666
Location	Aiguaviva	Ref	–	–
	Ordis	0.07	0.11	0.562
	Madremanya	0.08	0.10	0.427
	Vidreres	0.10	0.11	0.393
	Massanes	0.07	0.09	0.441
	Hostalnou de Bianya	-0.08	0.10	0.404
	Montagut	0.22	0.10	0.024
	St. Esteve de Llémena	-0.03	0.11	0.786
	Canet d'Adri	-0.02	0.11	0.891
	St. Feliu de Guíxols	0.16	0.15	0.308
	Riells i Viabrea	0.03	0.09	0.703
	Vilobí d'Onyar	-0.02	0.09	0.791
*L. infantum* seropositivity	Seronegative	Ref	–	–
	Seropositive	0.10	0.04	0.013

*Abbreviation*: *SE* standard error

^a^Level of significance of *P*-value < 0.05 was used

**Table 4:** Estimates of the multilevel linear regression model of the relationship between log transformed normalized rSP03B OD values and sampling time, location and dog seropositivity to *L. infantum*. “Dog” was included as a random effects variable

Variable	Levels	Estimate	SE	*P*-value^a^
Intercept		2.79	0.06	<0.001
Sampling month	February 2016	Ref	–	–
	August 2016	0.39	0.06	<0.001
	October 2016	0.09	0.03	0.003
	January 2017	-0.13	0.03	<0.001
	April 2017	-0.06	0.03	0.016
Location	Aiguaviva	Ref	–	–
	Ordis	0.06	0.10	0.563
	Madremanya	-0.04	0.09	0.652
	Vidreres	-0.03	0.10	0.783
	Massanes	0.05	0.08	0.533
	Hostalnou de Bianya	-0.16	0.09	0.074
	Montagut	0.18	0.09	0.035
	St. Esteve de Llémena	-0.10	0.10	0.287
	Canet d'Adri	-0.05	0.10	0.641
	St. Feliu de Guíxols	-0.19	0.14	0.173
	Riells i Viabrea	-0.08	0.08	0.302
	Vilobí d'Onyar	-0.06	0.08	0.399
*L. infantum* seropositivity	Seronegative	Ref	–	–
	Seropositive	0.07	0.03	0.032

*Abbreviation*: *SE* standard error

^a^Level of significance of *P*-value < 0.05 was used

## Discussion

The quantification of anti-sand fly saliva antibodies in vertebrate hosts of *L. infantum* has been previously shown to be an effective way of measuring exposure to the parasite vectors [16]. In the case of dogs, the most frequent host and reservoir of *L. infantum*, this has been proven for *P. perniciosus* [15, 26], as well as for other sand fly species [27–29]. These markers of exposure can then be applied in host-vector epidemiological studies, in *L. infantum* infection risk assessment, and to assist in the design of control strategies for the disease. Therefore, it is important to validate these techniques in natural, heterogeneous populations from endemic areas, in which a higher individual variability is expected.

*Phlebotomus perniciosus* activity period in Spain shows two main peaks, the first in June-July and the second in September-October. These peaks also correspond to the periods of highest *L. infantum* transmission [30–32]. This trend was identified in our study and corresponds to the rise in anti-saliva antibody levels observed between August and October. Humoral immune response to *P. perniciosus* saliva elicited in experimentally bitten dogs showed that antibody levels significantly rose after 2–4 weeks of continued exposure, peaking in week 5 [15]. In our field study, the highest IgG levels were in August, which clearly corresponded to the June-July *P. perniciosus* expected activity peak. Similarly, the high IgG readings obtained in October are likely to correspond to *P. perniciosus* second peak of activity. The lower rise in antibody levels observed at this time point can be explained by an earlier sampling at the beginning of October, which may have hindered the display of a complete seroconversion. The high overall levels of seropositivity to anti-sand fly saliva antigens, especially for SGH (49.43%), strongly support the CanL endemicity status for the region [33]. These results also validate both SGH and rSP03B as suitable antigens to assess exposure to *P. perniciosus* in natural canine populations from endemic areas.

An important remark when analysing the longitudinal dynamics of anti-sand fly saliva IgG in the study dog population is that there was a clear basal antibody level before the transmission season. After the expected rise in humoral response during summer months, IgG levels returned again to basal levels. These results show that, though exposed to repetitive bites during several months, dogs from endemic areas do not sustain high anti-saliva IgG levels throughout the year, allowing the detection of recent exposure to sand flies in natural populations. Similar results were recently reported in a longitudinal field study in Brazil, where canine IgG against *Lutzomyia longipalpis* saliva were evaluated [34]. Our study identified the same trends for both SGH and rSP03B, which reinforces the suitability of recombinant antigens in detecting recent exposure to phlebotomine vectors in endemic settings, particularly when considering the use of these tests in large-scale studies for vector control interventions [35, 36].

Antibodies recognizing both SGH and rSP03B followed similar dynamics throughout the field study. However, the correlation between the two antigens was only moderate (*r*_*S*_ = 0.54; 95% CI: 0.48–0.60, *P* < 0.001). Even so, available studies show that rSP03B is the most promising surrogate for SGH as a marker of exposure to *P. perniciosus* in the canine host. It has presented high levels of correlation with SGH in both experimentally [25] and naturally bitten dogs [16, 17, 26]. Two apyrase proteins (rSP01B and rSP01) have also shown a good correlation with SGH [25]. However, in a study where these three recombinant proteins presented similarly high correlations with SGH, rSP03B presented the lowest data dispersion and was considered a better option [16]. These results were confirmed in a field trial, where single rSP03B demonstrated a higher correlation coefficient with SGH than the combination of rSP03B with rSP01 [17].

A similar correlation between SGH and rSP03B to the one obtained in the present study has been observed before in Umbria region (central Italy) (*r*_*S*_ = 0.56; 95% CI: 0.38–0.71, *P* < 0.001; *n* = 96), in a screening study of dog exposure to *P. perniciosus* across European CanL endemic foci [26]. A possible reason for these discordant results may be the presence of other closely related phlebotomine species which could induce cross-reactivity with the SGH [22]. In some parts of Catalonia, *P. perniciosus* is sympatric with *P. ariasi*, also a proven vector of *L. infantum* [10]. Due to the close relationship between *P. perniciosus* and *P. ariasi*, both belonging to the subgenus *Larroussius*, it is expected that they share similar salivary antigens [37]. When comparing the percentage of seropositive dogs detected by both methods during the study, results for SGH are higher (49.43%) than for rSP03B (28.98%). Also, median results per sampling location show differences between SGH and rSP03B: in some cases, the trend between antigens is very similar (e.g. sera from Sant Feliu de Guíxols); in other cases, there is a recognizable peak in anti-SGH IgG, while anti-rSP03B IgG shows no change (e.g. sera from Madremanya). These differences can also be observed over time in the same location, with humoral responses to SGH and rSP03B peaking in different months along the transmission season (e.g. Canet d’Adri). We may hypothesize that SGH, because it contains more proteins than the single-antigen rSP03B, will more likely cross-react with antibodies against *P. ariasi*, inducing a stronger unspecific reaction to this vector species. It would also mean that the prevalence of sand fly species responsible for *L. infantum* transmission in the province varies according to the location, and possibly in the same location throughout the transmission season, for which it would be interesting to perform further entomological studies in the region.

Correlation indexes between levels of antibodies against both salivary antigens and *L. infantum* infection were low [SGH: *r*_*S*_ = 0.27 (95% CI: 0.19–0.35, *P* < 0.001); rSP03B: *r*_*S*_ = 0.25 (95% CI: 0.18–0.32, *P* < 0.001)]. Similar low correlations have been described before between sand fly bites and human visceral leishmaniasis (VL), while stronger correlations are reported between human cutaneous leishmaniasis (CL) and recent vector exposure (reviewed in [23]). This can be explained by VL’s longer incubation period and/or the differences in host immune responses to cutaneous and visceral infection [38]. Results from some studies in human populations also suggest that the repeated contact with non-infected sand flies could be correlated with markers of protection for VL [39]. Partial protection against *L. major*, an agent of CL, has also been achieved in immunized mice by the bites of uninfected sand flies [40]. However, another study with BALB/c mice demonstrated that this type of immunity is limited to short-term exposure and questioned the efficacy of sand fly saliva-induced protection against *Leishmania* infection in CL endemic areas [41]. CanL follows a pattern which is more similar to VL than to CL, therefore a low correlation between humoral responses to sand fly saliva and *Leishmania* would be expected [15]. However, results of the multilevel linear regression model show a positive and statistically significant relationship between *P. perniciosus* bites and a seropositive status for *L. infantum*, both for SGH and rSP03B. Similar results have been described in other longitudinal field studies on both canine anti-*P. perniciosus* and anti-*L. longipalpis* IgG dynamics [17, 34]. Unlike cross-sectional surveys, longitudinal studies are able to detect the relationship between a higher number of sand fly bites at a given time point and a subsequent *L. infantum* infection. Therefore, this type of study is likely to better explain the relationship between these two events, which can take place several months apart.

## Conclusions

The results of this study confirmed the applicability of both anti-*P. perniciosus* SGH and rSP03B IgG as markers of exposure to *L. infantum* vectors in natural dog populations from an endemic area. Canine humoral response to both antigens is compatible with the annual sand fly activity dynamics expected for the region. Significantly lower IgG levels were observed during the non-transmission season; despite the repeated exposure to sand flies during the summer months, there is a return to basal IgG levels in these dog populations during the winter. The comparative performance of SGH and rSP03B showed a moderate correlation, which might be explained by the occurrence of cross-reactions of SGH with other closely related sympatric sand flies. Further longitudinal studies in natural canine populations from endemic areas, together with entomological studies, should be carried out in order to corroborate this hypothesis. Nevertheless, both antigens are expected to detect only vectors of *L. infantum*, confirming their suitability for host-vector-parasite studies. Finally, the overall results support the CanL endemicity status for the study region, which had already been suggested by previous studies [33].

## Methods

### Experimental design

The study included a heterogeneous population of 176 dogs distributed by 12 locations in Girona Province (Catalonia, northeast of Spain), an area endemic for CanL [33]. These dogs were enrolled in a canine leishmaniosis vaccine field trial, but no statistically significant differences in *L. infantum* infection between groups were observed either during or at the end of the trial. These were all owned dogs, used mainly for hunting, but some breeding and racing individuals were also included. All animals were kept in large packs in open-air facilities, mostly in rural and periurban areas. Furthermore, no specific anti-sand fly insecticide treatments were applied, providing conditions for dog exposure to the vector. Dog density per study location varied between 4–23. The dogs were followed from February 2016 to April 2017 and blood samples, obtained by venepuncture and placed in 5 ml EDTA tubes, were collected at 5 pre-determined time points (Table 1). Plasma was obtained and stored at -40 °C until processing.

### Sand flies and salivary proteins

A colony of *P. perniciosus* was reared under standard conditions as described previously [42]. Salivary glands were dissected from 4–6 day-old females, pooled at a concentration of 1 salivary gland per 1 μl of 20 mM Tris buffer with 150 mM NaCl and stored at -80 °C. The *P. perniciosus* 43 kDa yellow-related recombinant protein (rSP03B, Genbank accn. DQ150622) was obtained from Apronex s.r.o. (Prague, Czech Republic) and quantified by the Lowry method (Bio-Rad, Hercules, California, USA) following the manufacturer’s protocol.

### Serological detection of dog exposure to sand flies

Anti-*P. perniciosus* IgG was measured by an in-house enzyme-linked immunosorbent assay (ELISA) as described previously [17]. All samples from a single dog were processed in the same plate. Briefly, microtiter plates were coated either with salivary gland homogenate (SGH) (40 ng per well, equivalent to 0.2 salivary gland) or with rSP03B (5 μg/ml) in 20 mM carbonate-bicarbonate buffer (pH 9.5) and incubated overnight at 4 °C. Plates were then blocked with 6% (w/v) low fat dry milk in PBS with 0.05% Tween 20 (PBS-Tw). Canine plasma were diluted 1:200 for SGH and 1:100 for rSP03B in 2% (w/v) low fat dry milk/PBS-Tw. Secondary antibodies (anti-dog IgG, Bethyl laboratories) were diluted 1:9000 in PBS-Tw. The reaction was stopped with 10% H_2_SO_4_ and absorbance was measured at 492 nm using a Tecan Infinite M200 microplate reader (Tecan, Männedorf, Switzerland). Each sample was tested in duplicate and positive and negative controls were included in each plate. To account for the variability between plates, sample OD readings were normalized by dividing them by the mean OD of positive controls run in the same plate [43]. The normalized OD values were multiplied by 100. Positivity cut-offs were calculated as the mean plus 3 standard deviations from 14 dog samples from a non-endemic area.

### Serological detection of *L. infantum* infection

All samples were tested for the presence of IgG against *L. infantum* through an in-house enzyme-linked immunosorbent assay (ELISA), using a technique described previously [44, 45]. Again, serial samples from a single dog were tested in parallel on the same plate. Briefly, dog plasma samples diluted at 1:400 were incubated in titration plates (Costar® Corning®, New York, USA) previously coated with sonicated whole promastigotes at a protein concentration of 20 μg/ml in 0.05 M carbonate buffer at pH 9.6. Protein A peroxidase (1:30,000, Sigma-Aldrich®, St. Louis, Missouri, USA) was used as conjugate and reactions were stopped with H_2_SO_4_ 3M when a pre-determined calibrator control serum reached an optical density of 450 at 450 nm. Sample optical densities were read at 492 nm. All samples were run in duplicate and the calibrator, positive and negative sera were included in all plates. Results were expressed in standard units (U) compared to a calibrator control sample set arbitrarily at 100U. The positivity cut-off was established at 24U.

### Statistical analysis

Statistical analyses were performed using R software (http://cran.r-project.org/) and Stata 15 software (StataCorp LP, College Station, TX, USA).

Correlations between IgG responses to *P. perniciosus* SGH and rSP03B and between each one of the salivary antigens and anti-*L. infantum* IgG levels were tested by the Spearman rank correlation test. Median OD values between time points were compared using the Wilcoxon signed rank sum test.

The relationship between anti-SGH and anti-rSP03B antibodies and sampling month, *L. infantum* infection status and location was tested by fitting multilevel linear regression models, taking into account the correlation between repeated measures of the same dogs over time. In the models, log-transformed anti-saliva or rSP03B normalized OD values were considered as continuous dependent variables and sampling month, *L. infantum* infection and location as categorical predictor variables. In order to assess variations in OD between the first sampling month and those following, “February 2016” was set as reference level for this variable. Likewise, the locality with the lowest median OD (“Aiguaviva”) was considered to be the reference for the variable location. Finally, “seronegative” was set as the reference level for the variable *L. infantum* infection. The random component included dog and time to allow for variation at the intercept (between dogs) and the slope (over time). The inclusion of “dog” as a random effects variable significantly improved both models, with a between dog variance of 48% for SGH and of 47% for the rSP03B model. A *P*-value of < 0.05 was considered to indicate statistical significance.

## Abbreviations

CanL: Canine leishmaniosis
CL: Human cutaneous leishmaniasis
ELISA: Enzyme-linked immunosorbent assay
IgG: Immunoglobulin G
OD: Optical density
rSP03B: 43 kDa yellow-related recombinant protein
SGH: Salivary gland homogenate
VL: Human visceral leishmaniasis
